# Instant Abdominal Wall Reconstruction with Biologic Mesh following Resection of Locally Advanced Colonic Cancer

**DOI:** 10.1155/2012/959342

**Published:** 2012-04-22

**Authors:** Oskay Kaya, Engin Olcucuoglu, Gaye Seker, Hakan Kulacoglu

**Affiliations:** Department of General Surgery, Diskapi Yildirim Beyazit Teaching and Research Hospital, 06810 Ankara, Turkey

## Abstract

We present a case of immediate abdominal wall reconstruction with biologic mesh following the resection of locally advanced colonic cancer. The tumor in the right colon did not respond to neoadjuvant chemotherapy. Surgical enbloc excision, including excision of the invasion in the abdominal wall, was achieved, and the defect was reconstructed with porcine dermal collagen mesh. The patient was discharged with no complication, and adaptation of the mesh was excellent at the six-month followup.

## 1. Introduction

Complex abdominal wall defects represent a reconstructive challenge to surgeons. Substantial empirical evidence suggests that the use of prosthetic mesh for the closure of abdominal wall defects is associated with significantly reduced cumulative recurrence rates after short- and long-term followup. However, mesh repair of the abdominal wall defect, particularly synthetic mesh repair, is associated with several postimplantation complications, such as wound infections, mesh infections, bowel adhesions, and other complications frequently requiring revision surgery [1]. When repairing an abdominal wall defect, a prosthetic mesh sometimes needs to be placed directly on the parietal peritoneum. Although the standard mesh for this purpose is laminar implant expanded polytetrafluoroethylene (PTFE), it is gradually being replaced by laminar collagen-based mesh [2]. In the literature, there are some case reports of the abdominal wall being reconstructed with biologic mesh after surgical excision of the area invaded by malignant tumor cells [3].

We herein present a case of immediate abdominal wall reconstruction with porcine dermal collagen mesh following the resection of locally advanced colon cancer.

## 2. Case Presentation

**Figure 1:**
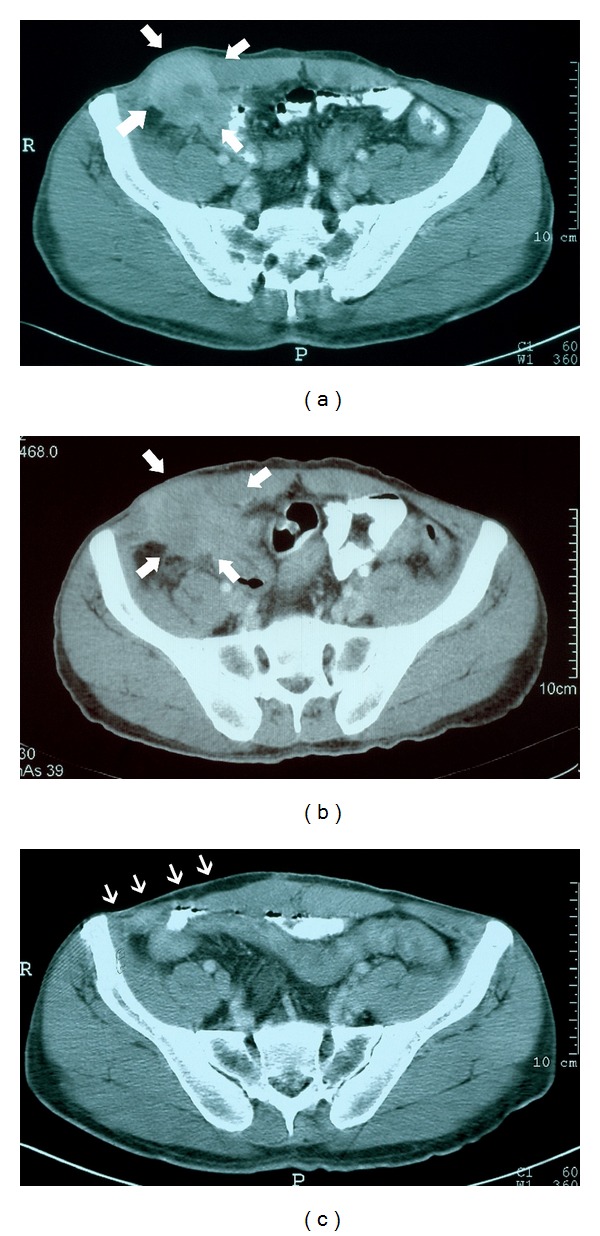
(a) The first CT of the patient before neoadjuvant chemotherapy. Thick arrows show the colonic mass, which invaded adjacent tissues including skin. (b) There was not enough mass regression after neoadjuvant therapy. (c) Excellent mesh adaptation at 6 months. Thin arrows highlight the mesh area.

A 42-year-old man admitted to the hospital complaining of a painful abdominal mass and pain in the right lower quadrant. There was also serous-purulent discharge through the skin incision over the mass. He had undergone appendectomy via a McBurney incision and an umbilical hernia repair via midline incision 4 years previously. Histopathology had revealed inflammatory changes with no additional abnormalities. Abdominal examination revealed an abdominal mass in the right lower quadrant. Laboratory data and tumor markers were within normal limits. Wound culture results over the course of one year showed negative discharge. He declined to undergo the surgical exploration suggested at a local hospital and was referred to our tertiary reference hospital. Ultrasound revealed a solid abdominal mass that was approximately 5 cm in diameter, localized near the cecum and the skin incision. There were mesenteric reactive nodes, 20 mm in maximum diameter. No sign of hernia was recorded upon performance of the Valsalva maneuver. An abscess with dense contents was observed. Computed tomography scan (CT) confirmed the existence of a solid tumor 7 cm in diameter, originating from the cecum and ileocecal valvula, invading the anterior abdominal wall and skin. There were pericolonic, paraaortic, and paraceliac lymph nodes of 2.5 cm in maximum diameter (Figure 1(a)). Colonoscopic investigation revealed an ulcero-vegetative mass filling the bottom of the cecum. Multiple biopsies were obtained, and histopathology revealed adenocarcinoma. The medical oncology department advised a neoadjuvant FOLFOX regimen. The patient could not be given the last cycle of the treatment because of increasing wound discharge and fever. Repeat CT demonstrated an inadequate response to chemotherapy (Figure 1(b)). The patient was then referred back to the surgical department.

**Figure 2:**
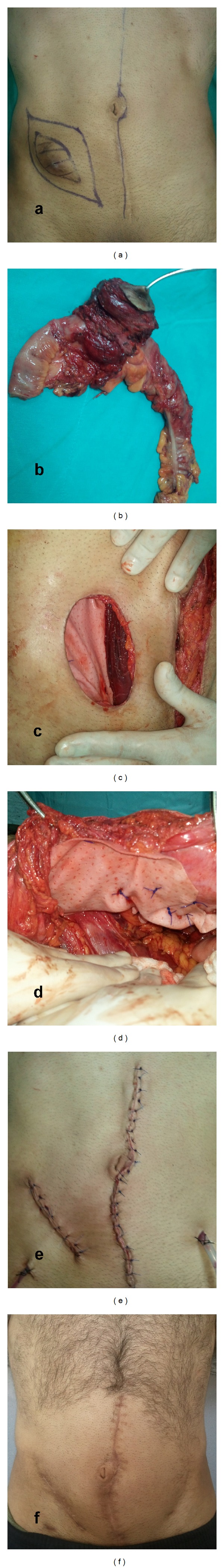
(a) Incisions were marked before surgery. (b) En-bloc resection material after extended right hemicolectomy. (c) Mesh applied to cover the abdominal wall defect, external view. (d) The mesh was secured with interrupted polypropylene sutures from inside. (e) The surgery has been completed and the skin closed. (f) Late postoperative view of the abdomen.

 Wide excision of the tumor was planned. A cystoscopic examination was performed, and a double-J catheter was applied to the right ureter to facilitate a safe surgical dissection. The mass and possible intraabdominal margins were marked before the operation (Figure 2(a)). Preoperatively, single dose antibiotic prophylaxis was applied. Laparotomy revealed a colonic mass invading the anterior abdominal wall and skin. En bloc resection of the lesion was achieved (Figure 2(b)). Between the skin and peritoneum macroscopically tumour invaded tissues including skin, subcutaneal adipose tissues, muscles, and fascias were excised with secure margins while performing standard right hemicolectomy. After lymphatic dissections were completed, end-to-side ileotransversostomy was done via two layers of suturing. The abdominal wall defect was reconstructed with 20 cm × 10 cm and 1.5 mm thickness porcine dermal Permacol mesh (Covidien Ltd. Dublin, Ireland; Mansfield, Massachusetts, US). Mesh fixation was performed with 2/0 prolen separate sutures on the inside of safe abdominal wall (Figures 2(c) and 2(d)). After surgical procedure two suction drains were applied before closure of abdomen. One was placed on the right lower quadrant, over the mesh in the reconstructed abdominal wall defect area, and the other one was placed on the left symmetrical location in the peritoneal cavity (Figure 2(e)). No blood transfusion was required. Histopathology revealed a mildly differentiated adenocarcinoma of 11 cm maximum diameter. It had invaded the dermis but not the epidermis. There was vascular but not neural invasion. Only one lymph node out of a total of 20 lymph nodes retrieved was found to have metastasized tumor cells. Peritoneal cytology was negative. The case was determined as Stage 3-C according to the TNM system. After beginning of intestinal motility, the patient was given oral intake on the second postoperative day. Intraperitoneal drain was removed at the same time. He was discharged after a four-day uneventful stay. Only simple analgesics were prescribed. The drainage volume from the first drain was measured daily. It was 100 cc and serous on first postoperative day and gradually reduced to 10 cc/day on seventh day. Then it was removed. The monthly follow up observations were planned. There was neither sign nor symptom of local inflammation for six months. The patient complained of mild pain and paresthesia in the right lower quadrant and right upper leg during the first month of followup. His neurologic examination was normal. There was no abnormal finding on lumbar magnetic resonance imaging and electromyography. Local ultrasound investigation of the wound revealed no collection. We observed excellent mesh adaptation with physical and CT examination at six months postoperatively (Figures 2(f) and 1(c)).

## 3. Discussion

As herein defined there was a large abdominal wall defect. Primary closure was impossible, and we had to use a prosthetic material. Although the efficacy of various biologic meshes in the abdominal wall reconstruction of complex ventral hernia has been shown, the performance profile of various biologic mesh scaffolds in terms of hernia-specific outcomes such as recurrence, mesh explantation, and mesh infections has not been examined sufficiently. Permacol Biologic Implant (PC) is a porcine dermal collagen implant from which cells, DNA and RNA, are removed in a gentle process that is not damaging to the 3D collagen matrix. The resulting acellular collagen matrix is then cross-linked for enhanced durability in complex repairs [4]. We chose this material to reconstruct the abdominal wall defect.

 Human-derived, porcine cross-linked, and non-cross-linked porcine bioprosthetic materials were compared in Shah et al.'s retrospective study to evaluate the clinical outcomes of patients who underwent complex ventral hernia repair with bioprosthetic material. The authors recorded at least one complication in 72.4% of the patients [1]. Harth and Rosen reported some major complications associated with xenograft biologic mesh implantation in abdominal wall reconstruction. They retrospectively reviewed an FDA database for reported xenograft-associated adverse events. They found that cross-linked meshes were associated with the most adverse event reports to the FDA and concluded that the findings from that FDA database review pointed toward a need to carefully evaluate these products [5]. In our case there was no problem about application of cross-linked porcine dermal graft in early postoperative period and after six months follow up.

Orenstein et al. demonstrated that porcine-derived meshes induce monocyte/macrophage activation in vitro. However, chemically crosslinked dermis induced significantly higher cytokine expression compared to non-cross-linked dermis [6]. Jarman-Smith and coworkers investigated porcine collagen cross-linking, degradation, and the capability for fibroblast adhesion and proliferation. They found that the resistance of the matrix to degradation by collagenases was gained via chemical crosslinking [7]. Deeken and his coworkers designed a study to compare the histologic and biochemical evaluation of crosslinked and non-cross-linked biologic meshes in a porcine model of ventral incisional hernia repair. They found that the tensile strengths of sites repaired with biologic mesh were not impacted by very high de novo tensile strength/stiffness or mesh-specific variables such as crosslinking. Although crosslinking distinguishes biologic meshes in the short-term based on the comparison of histological features, such as cellular infiltration and neovascularization, many differences diminish over longer periods of time. Characteristics other than crosslinking, such as tissue type and processing conditions, are likely responsible for these differences [8]. Similar results were found in Melman et al.'s study [9]. In another prospective pilot study, Hammond and coworkers investigated the human in vivo cellular response to cross-linked acellular collagen implants. They studied tissue biopsies obtained from implants and found that this type of implant had excellent potential for tissue reinforcement [10]. We had to remove a large field of abdominal wall including all layers from skin to peritoneum. Because of this reason prosthetic material that we thought to apply might exactly touch to intraperitoneal structures, and we worried about probable adhesions and their bad results. Theoretical and practical advantages of PC were well established in the literature [11, 12]. This material supports growth and fibrinolytic activity of human mesothelial cells; also this material is well tolerated as a subcutaneous implant with only a minor chronic inflammatory response. All these positive effects of PC mesh make us to choose it to repair the large abdominal defect of our case. Porcine-derived biomaterials have typically been used to repair ventral hernias in clinic and experimental studies [13].

 Abdominal compartment syndrome is a serious life-threatening condition observed after abdominal closure in patients with severe wall defects. Similar conditions must be mentioned in pediatric organ transplant recipients. In literature PC was used to gain extra abdominal volume and to prevent the syndrome in these conditions [14]. In our case the abdominal wall defect was not suitable for primary closure; therefore PC was used to avoid abdominal compartment syndrome.

The bacterial clearance of biologic grafts used in hernia repair was investigated in a rat model. It was found that biologic grafts, compared to synthetic material, were better able to clear a *Staphylococcus aureus* contamination [15]. Biologic graft was preferred in a subsequent definitive abdominal closure after an apronectomy performed for damage control in a patient with necrotizing fasciitis and strangulated umbilical hernia [16]. PC has also been used for hernia repair in contaminated fields [17]. Our presented patient has serous-purulent discharge over the mass. Even though the wound culture results were negative, we preferred to use biologic graft in concerning contamination.

 Notably, the treatment of abdominal wall neoplasm, whether primary or secondary, continues to present a challenging problem. Treatment typically involves massive soft-tissue loss; thus, this highly aggressive resection sometimes generates a large, complex abdominal wall defect. Also, there is a need for immediate reconstruction, if the vital structures are exposed. Synthetic mesh such as polypropylene (PP) is commonly used to repair other trunk defects. As a result of its macroporous structure, the mesh induces intense fibrovascular infiltration and incorporates into the surrounding myofascial tissue to provide a strong repair. However, it is also associated with adhesions to intra-abdominal viscera and enterocutaneous fistula formation. Furthermore, it is largely intolerant to infection, particularly if used in contaminated or infected fields or in the event of exposure as a result of dehiscence or breakdown of the overlying skin. Abdominal wall tumors sometimes accompany contamination or infection. Such situations contraindicate implanting synthetic mesh. In a prospective study, Tang and coworkers investigated the immediate repair of major abdominal wall defects after extensive tumor excision in patients with abdominal wall neoplasm. The authors studied 27 cases, and they concluded that biological mesh was an ideal alternative to synthetic mesh for abdominal wall restoration after tumor resection, especially in situations of infection or contamination [18]. In another study, a total of 30 patients underwent reconstruction after full-thickness resection of abdominal wall tumors or tumors of intra-abdominal organs involving the abdominal wall. Acellular dermal matrix was used more commonly in the patients with tumors of gastrointestinal origin; it has been a useful tool to minimize morbidity and recurrence in these high-risk patients [19]. Ghazi et al. retrospectively reviewed all patients who required the reconstruction of complex abdominal wall defects (165 patients in seven years). Mesh was used in 81.8% of cases, 77% of those being acellular dermal matrices. The authors found that the recurrence rate was similar for synthetic and biomesh reconstructions; however, the complication rates were higher when synthetic mesh was used [20].

 Our presented case was locally advanced colon cancer with invaded abdominal wall. Our first experience showed that, according to the results of observations, biologic meshes can safely be used in repair of large and probable contaminated abdominal wall defects.
